# Collaborating with Patients and Communities to Identify Healthy Reentry Needs of Older Women after Incarceration

**DOI:** 10.1093/geroni/igaf122.3899

**Published:** 2025-12-31

**Authors:** Amanda Emerson, Jill Peltzer, Amanda Thimmesch, Alexandria Thompson, Bernard Schuster, Sharla Smith, Janet Severine

**Affiliations:** University of Kansas Medical Center, Kansas City, Kansas, United States; University of Kansas Medical Center, Kansas City, Kansas, United States; University of Kansas Medical Center, University of Kansas Medical Center, Kansas, United States; University of Kansas Medical Center, Kansas City, Kansas, United States; University of Kansas Medical Center, Kansas City, Kansas, United States; University of Kansas Medical Center, Kansas City, Kansas, United States; University of Kansas Medical Center, Kansas City, Kansas, United States

## Abstract

**Background:**

Older women’s health needs after incarceration have not been a focus in research, resulting in a lack of evidence to guide the design of programs to support health in reentering older women. In Older Women Leading Healthy Aging Research Together (OWLHART), our Kansas City-based team worked with a patient-based community group to answer two questions: “What are priority health needs for older women after incarceration?” and “What are opportunities and obstacles to supporting older women in healthy aging after incarceration?”

**Method:**

With the 12-member OWLHART Network, we planned listening sessions and developed interview guides. Group and individual interviews took place on Zoom with three participant groups in February 2025: older women with lived experience of incarceration (LEI), community and peer advocates, and healthy aging researchers. OWLHART then joined us over several months in thematically analyzing deidentified data using the framework method and comparing themes across groups.

**Results:**

Five individual or group interviews were conducted with seven women (age 48-68) with LEI, four community advocates, and two researchers. Theme clusters included “priority health-related needs of older women during reentry”; “solutions that do or could work”; and “what’s research got to do with it.” Convergence was observed across the three clusters with notable divergences in priority needs and research.

**Conclusion:**

Women, advocates, and researchers mostly agree about priority needs and what works in supporting older women post-incarceration. Future intervention and health promotion design would benefit from leveraging older women’s desire to be involved in developing and implementing solutions.

